# *Mycobacterium abscessus* ssp. *abscessus* infection progressing to empyema from vertebral osteomyelitis in an immunocompetent patient without pulmonary disease: a case report

**DOI:** 10.1186/s12890-019-0860-4

**Published:** 2019-05-24

**Authors:** Naoki Kadota, Tsutomu Shinohara, Hiroyuki Hino, Yuichiro Goda, Yoshiro Murase, Satoshi Mitarai, Fumitaka Ogushi

**Affiliations:** 1 Division of Pulmonary Medicine, National Hospital Organization Kochi Hospital, 1-2-25 Asakuranishimachi, Kochi, 780-8077 Japan; 2 Department of Clinical Investigation, National Hospital Organization Kochi Hospital, 1-2-25 Asakuranishimachi, Kochi, 780-8077 Japan; 3 Division of Thoracic Surgery, National Hospital Organization Kochi Hospital, 1-2-25 Asakuranishimachi, Kochi, 780-8077 Japan; 4 Division of Orthopaedic Surgery, National Hospital Organization Kochi Hospital, 1-2-25 Asakuranishimachi, Kochi, 780-8077 Japan; 5 Department of Mycobacterium Reference and Research, Research Institute of Tuberculosis, Japan Anti-Tuberculosis Association, 3-1-24 Matsuyama, Kiyose, Tokyo 204-8533 Japan

**Keywords:** *Mycobacterium abscessus* ssp. *abscessus*, Vertebral osteomyelitis, Empyema

## Abstract

**Background:**

Pleural involvement by non-tuberculous mycobacteria (NTM) in patients without distinct pulmonary disease is extremely rare. Vertebral osteomyelitis (VO) with or without pulmonary disease is also a rare clinical presentation of NTM infection, and pleural spread of NTM from VO has not been reported.

**Case presentation:**

A 63-year-old woman was admitted to our hospital with back pain persisting for 4 months and a 2-day history of fever and right chest pain. The patient was initially treated as right-sided empyema due to general bacteria. However, after removal of the chest tube, a previously overlooked paravertebral lesion was observed on CT. MRI confirmed VO at T7/8. *Mycobacterium abscessus* ssp. *abscessus* was detected in both the thoracic cavity and the paravertebral lesion. Both VO and the paravertebral abscess were improved by antimycobacterial treatment.

**Conclusion:**

VO of the thoracic spine due to non-tuberculous mycobacterial infection should be considered as a cause of pleuritis or empyema without pulmonary disease, especially in patients with back pain.

## Background

*Mycobacterium abscessus (M. abscessus)* ssp. *abscessus*, a rapidly growing species of non-tuberculous mycobacteria (NTM), is well-known as a pathogen of the skin, soft tissues, bone, and lungs [1–3]. NTM, especially *M. avium-intracellulare* complex (MAC), occasionally causes pleuritis or empyema, probably due to direct spread from pulmonary lesions [4–9]. However, pleural involvement without distinct pulmonary disease is extremely rare, with only a few cases in the literature, and primary pleural disease due to *M. abscessus* ssp. *abscessus* has not been reported [10–13].

Vertebral osteomyelitis (VO) with or without pulmonary disease is also a very rare clinical presentation of NTM infection, including that caused by *M. abscessus* complex [14–18]. Approximately half of all VO develops in immunocompetent patients and most frequently affects the thoracic spine [14]. According to a recent hypothesis, “locus minoris resistentiae” after noninvasive trauma may be a risk factor for VO, i.e., macrophages containing NTM migrate to the site of injury and release the mycobacteria to initiate a new focus of infection [16]. However, pre-disposing trauma or surgery is not reported in 85% of patients with VO [14]. Here we report a very unusual case of *M. abscessus* ssp. *abscessus* infection that presented as empyema without distinct pulmonary disease and was found to arise from VO.

## Case report

**Fig. 1:**
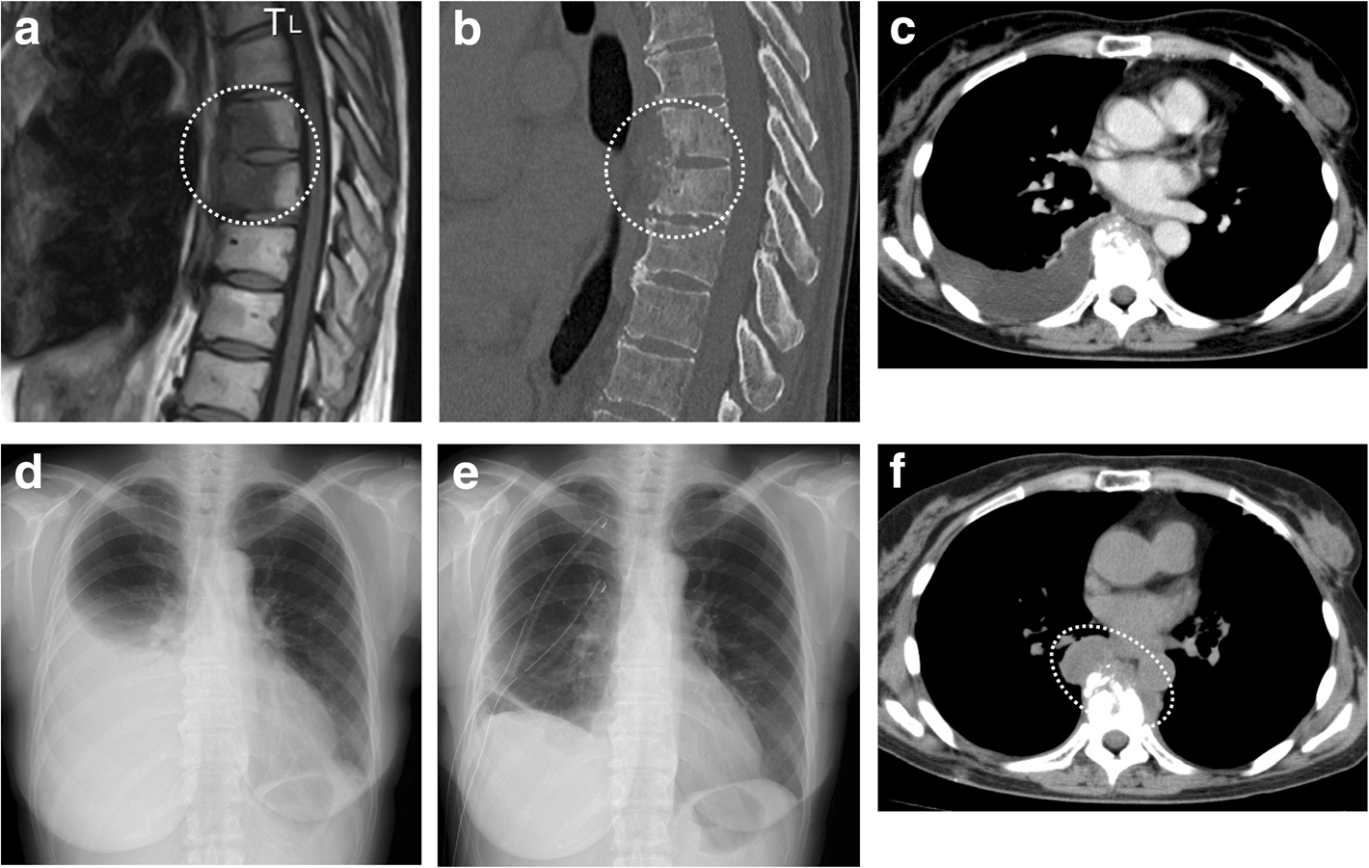
Imaging findings before antimycobacterial treatment (**a** T1-weighted spinal MRI obtained 2 months before admission, **b** spinal CT obtained 3 weeks before admission, **c** enhanced chest CT scan on admission, **d** chest X-ray film after treatment with ampicillin/sulbactam for 11 days, **e** chest X-ray film during chest drainage and administration of cefoperazone/sulbactam following thoracoscopic curettage, **f** chest CT scan one month after chest tube removal). Spinal MRI and CT detected a T7/8 vertebral lesion (circled). Chest drainage achieved satisfactory re-expansion of the right lung. After removal of the chest tube, a paravertebral lesion was detected on CT (circled)

A 63-year-old woman was admitted to our hospital with back pain persisting for 4 months and a 2-day history of fever and right chest pain. On admission, her height and weight were 154 cm and 50 kg, respectively. She had no history of other diseases, including autoimmune disease, diabetes, bronchiectasis, old healed tuberculosis, trauma, or acupuncture. The patient had visited two other hospitals, where contusion of the thoracic spine had been diagnosed by MRI (two months before admission) and contrast CT (three weeks before admission) (Fig. 1a, b), despite no history of trauma. She had received symptomatic therapy with an anti-inflammatory agent from both hospitals, but her back pain had persisted.

Initial laboratory data included a white blood cell count of 7580/μl (85.0% neutrophils) and a C-reactive protein of 8.26 mg/dl. CT showed a right-sided pleural effusion (Fig. 1c). Right pleuritis was diagnosed and the patient was treated with ampicillin/sulbactam for 11 days, but this was not effective (Fig. 1d). She subsequently underwent thoracoscopic curettage followed by drainage of pus from the pleural cavity for 7 days using 22 and 24 Fr double lumen trocars, and administration of cefoperazone/sulbactam for the same period (Fig. 1e). General bacterial culture of pus obtained at surgery was negative, but culture for acid-fast bacteria (mycobacteria growth indicator tube (MGIT) system; BACTEC MGIT 960) proved to be positive after the 7-day treatment period. The pathogen was identified as *M. abscessus* complex by DNA-DNA hybridization [19], and was confirmed to be *M. abscessus* ssp. *abscessus,* but not *M. abscessus* ssp. *massilense* or *M. abscessus* ssp. *bolletii* [20], by multiplex PCR [21] and *rpoB* sequence analysis [22]. Since there was no previous report of primary empyema due to *M. abscessus* ssp. *abscessus* and the patient had no underlying disease suggesting a source of infection, the result was considered to represent contamination and further treatment was not provided. However, CT performed one month later revealed progression of a previously overlooked paravertebral lesion to involve the lung (Fig. 1f). *M. abscessus* ssp. *abscessus* was detected from lavage fluid of the paravertebral lesion recovered by bronchoscopic examination. Two months after admission (5 weeks after initial detection of *M. abscessus* ssp. *abscessus*), treatment with imipenem/cilastatin (IPM/CS: 1 g/day i.v.), amikacin (AMK: 400 mg/day i.v.), and clarithromycin (CAM: 800 mg/day p.o.) was initiated based on a diagnosis of VO due to *M. abscessus* ssp. *abscessus,* with paravertebral abscess caused by direct spread. An antibiotic susceptibility test was performed with air-dried microplates containing serial dilutions of antimicrobial agents and modified Middlebrook 7H9 broth [23], revealing that the minimum inhibitory concentration (MIC) of CAM for the pathogen was 0.25 μg/ml on day 3 and 1.0 μg/ml on day 14. These data indicated that the pathogen remained susceptible to CAM (MIC ≤2.0 μg/ml on days 3 and day 14) and did not develop inducible resistance (susceptible on day 3 with MIC ≥8.0 μg/ml at day 14), according to the Clinical Laboratory Standards Institute Guideline [24, 25].

**Fig. 2:**
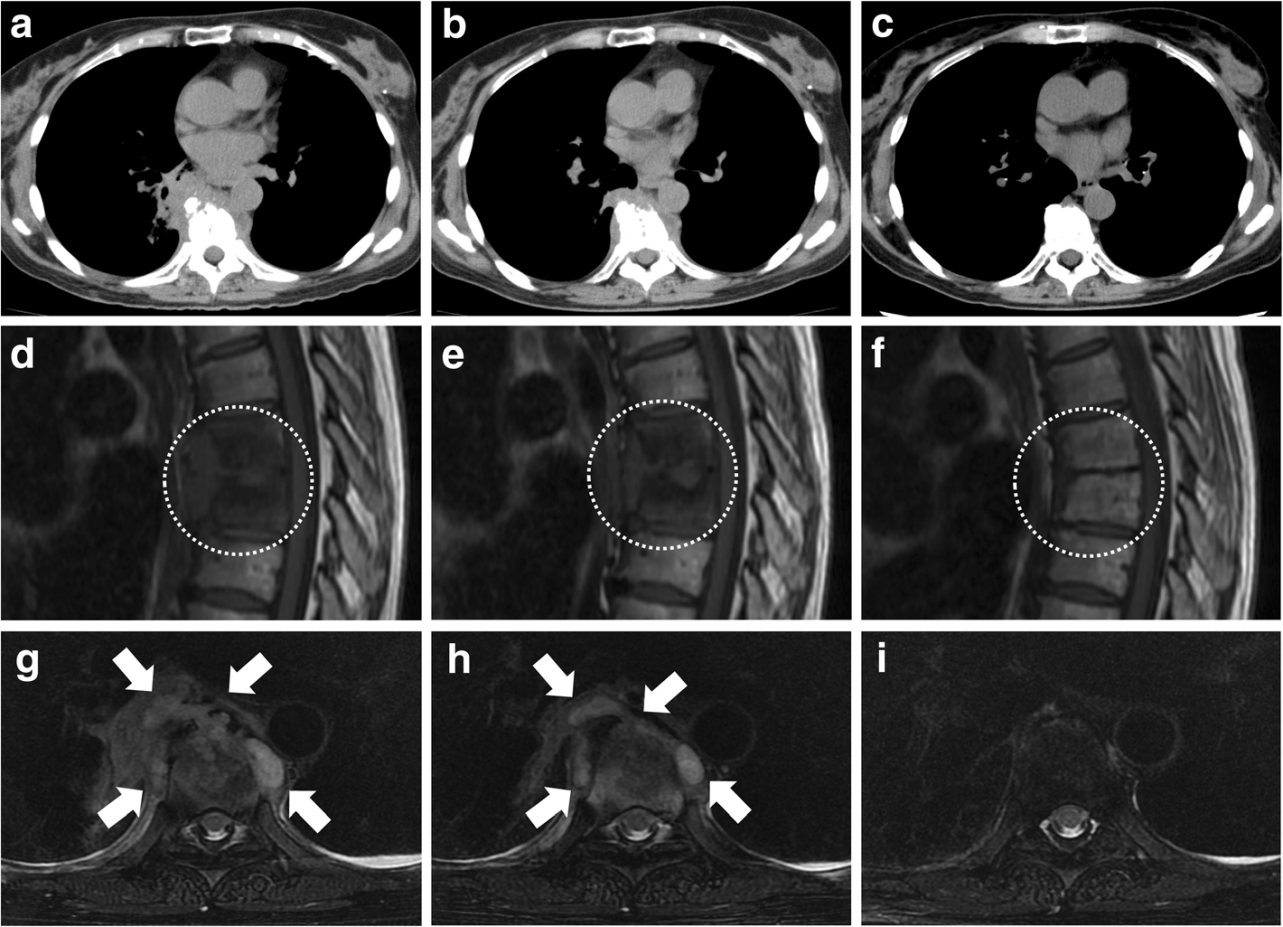
Chest CT scans (**a**-**c**) and spinal MRI (**d**-**f** T1-weighted, **g**-**i** fat-suppressed T2-weighted) obtained after initiation of antimycobacterial treatment (**a**, **d** and **g** at 1 month, **b**, **e** and **h** at 3 months, **c**, **f** and **i** at 2 years). Both the VO (bone destruction on CT and low signal on T1-weighted MRI (circled)) and the abscess (paravertebral lesion on CT and high signal on fat-suppressed T2-weighted MRI (arrow)) improved gradually over 2 years

After continuation of treatment for three months, both the MRI-confirmed VO and the paravertebral abscess showed improvement (Fig. 2a, b, d, e, g, h), so she was switched to oral antibiotic therapy (faropenem (FRPM) 600 mg/day, levofloxacin (LVFX) 500 mg/day, and CAM 800 mg/day). After that, further improvement was observed and the antimycobacterial treatment was completed within 2 years (Fig. 2c, f, i). No other combination therapy was administered during this period. No evidence of recurrence has been detected during follow-up for 4 months after the end of treatment.

## Discussion and conclusions

In patients with pleural involvement alone, the main pathway by which NTM reaches the thoracic cavity is thought to be direct discharge of an undetectable subpleural caseous focus into the pleural space [10]. However, early *M. abscessus* complex pulmonary disease is generally of the nodular bronchiectatic type, so a subpleural caseous focus is an uncommon incipient lesion of *M. abscessus* complex infection compared to MAC infection [26]. In our patient, the VO was considered to be the initial focus of infection for the following reasons. First, empyema occurred after vertebral abnormality was detected on imaging studies at previous hospitals. Second, *M. abscessus* ssp. *abscessus* was not only isolated from the thoracic cavity, but also from the paravertebral abscess. The reason for non-recurrence of empyema was considered to be postoperative pleural adhesion induced by thoracoscopic curettage and subsequent drainage. Third, although vertebral biopsy was not performed, the paravertebral abscess and vertebral lesion both improved simultaneously with antimycobacterial treatment, suggesting that VO was also caused by *M. abscessus* ssp. *abscessus* infection. According to a review of 13 cases of pyogenic spondylitis with exudative pleural effusion, *Staphylococcus aureus* was the major pathogen [27]. To the best of our knowledge, this is the first reported case of NTM with a similar clinical presentation to those cases. Although there was no clear history of pre-disposing trauma, “locus minoris resistantiae” may have been the mechanism of underlying VO, since our patient had no peripheral pulmonary lesion.

Her treatment (IPM/CS 1 g/day, AMK 400 mg/day and CAM 800 mg/day followed by FRPM 600 mg/day, LVFX 500 mg/day and CAM 800 mg/day) was selected on the basis of drug sensitivity data for *M. abscessus* ssp. *abscessus* obtained in Japanese patients and a report on a patient successfully treated with this regimen [3, 28, 29]. Standard criteria have not been established for terminating treatment of *M. abscessus* ssp. *abscessus* infection, but the patient preferred to stop medication when the lesion almost resolved on imaging. In recent years, it has been suggested that inducible and acquired resistance to CAM are the main causes of treatment-refractory *M. abscessus* ssp. *abscessus* pulmonary disease [25, 30–32]. Inducible macrolide resistance (susceptible on day 3 but resistant on day 14) is a natural trait of *M. abscessus* spp. *abscessus* due to ribosomal methyl transferase gene *erm*(41). However, T/C polymorphism occurs at position 28 of *erm*(41) and C28 strains usually lose *erm*(41) function, resulting in susceptibility to macrolides [32]. Although genetic analyses were not performed, the isolate from our patient was susceptible and did not show inducible resistance to CAM. On the other hand, acquired macrolide resistance develops during treatment, and is associated with mutations of the *rrl* gene region encoding the peptidyltransferase domain of the 23S rRNA [25, 32]. Because no acid-fast bacteria were detected in our patient after the start of macrolide treatment, it is unknown whether there was a change in susceptibility of *M. abscessus* ssp. *abscessus* to CAM. However, the good clinical course of our patient makes it unlikely that acquired resistance to CAM developed during the treatment period.

Delayed diagnosis of spinal disease may lead to neurologic complications. Accordingly, VO of the thoracic spine due to NTM infection should be considered as a cause of pleuritis or empyema in patients without pulmonary disease, especially when back pain is present.

## Abbreviations

AMK: Amikacin
CAM: Clarithromycin
FRPM: Faropenem
IPM/CS: Imipenem/cilastatin
LVFX: Levofloxacin
MAC: *Mycobacterium avium-intracellulare* complex
MIC: Minimum inhibitory concentration
NTM: Non-tuberculous mycobacteria
VO: Vertebral osteomyelitis

## References

[1] Piersimoni C, Scarparo C (2009). Extrapulmonary infections associated with nontuberculous mycobacteria in immunocompetent persons. Emerg Infect Dis.

[2] Daley CL, Griffith DE (2002). Pulmonary disease caused by rapidly growing mycobacteria. Clin Chest Med.

[3] Harada T, Akiyama Y, Kurashima A, Nagai H, Tsuyuguchi K, Fujii T (2012). Clinical and microbiological differences between *Mycobacterium abscessus* and *Mycobacterium massiliense* lung diseases. J Clin Microbiol.

[4] Park S, Jo KW, Lee SD, Kim WS, Shim TS (2017). Clinical characteristics and treatment outcomes of pleural effusions in patients with nontuberculous mycobacterial disease. Respir Med.

[5] Yanagihara K, Tomono K, Sawai T, Miyazaki Y, Hirakata Y, Kadota J (2002). *Mycobacterium avium* complex pleuritis. Respiration..

[6] Park SU, Koh WJ, Kwon OJ, Park HY, Jun HJ, Joo EJ (2006). Acute pneumonia and empyema caused by *Mycobacterium intracellulare*. Intern Med.

[7] Asai K, Urabe N (2011). Acute empyema with intractable pneumothorax associated with ruptured lung abscess caused by *Mycobacterium avium*. Gen Thorac Cardiovasc Surg.

[8] Olafsson EJ, Naum CC, Sarosi GA, Mastronarde JG (2004). Bilateral pleural effusions and right pneumothorax in a 25-year-old man. Chest..

[9] Lee YC, Kim SB, Gang SJ, Park SY, Kim SR (2015). Acute necrotizing pneumonia combined with parapneumonic effusion caused by *Mycobacterium lentiflavum*: a case report. BMC Infect Dis.

[10] Ikeue T, Yoshida H, Tanaka E, Ohi I, Noguchi S, Fukao A (2017). Pleuritis caused by *Mycobacterium kyorinense* without pulmonary involvement. Intern Med.

[11] Okada Y, Ichinose Y, Yamaguchi K, Kanazawa M, Yamasawa F, Kawashiro T (1995). *Mycobacterium avium-intracellulare* pleuritis with massive pleural effusion. Eur Respir J.

[12] Nagaia T, Akiyama M, Mita Y, Tomizawa T, Dobashi K, Mori M (2000). Mycobacterium avium complex pleuritis accompanied by diabetes mellitus. Diabetes Res Clin Pract.

[13] Fabbian F, De Giorgi A, Pala M, Fratti D, Contini C (2011). Pleural effusion in an immunocompetent woman caused by *Mycobacterium fortuitum*. J Med Microbiol.

[14] Kim CJ, Kim UJ, Kim HB, Park SW, Oh MD, Park KH (2016). Vertebral osteomyelitis caused by non-tuberculous mycobacteria: predisposing conditions and clinical characteristics of six cases and a review of 63 cases in the literature. Infect Dis (Lond).

[15] Sarria JC, Chutkan NB, Figueroa JE, Hull A (1998). Atypical mycobacterial vertebral osteomyelitis: case report and review. Clin Infect Dis.

[16] Chan ED, Kong PM, Fennelly K, Dwyer AP, Iseman MD (2001). Vertebral osteomyelitis due to infection with nontuberculous Mycobacterium species after blunt trauma to the back: 3 examples of the principle of locus minoris resistentiae. Clin Infect Dis.

[17] Garcia D. C., Sandoval-Sus J., Razzaq K., Young L. (2013). Vertebral osteomyelitis caused by Mycobacterium abscessus. Case Reports.

[18] Edwards C, Diveronica M, Abel E (2012). Epidural abscess caused by *Mycobacterium abscessus*. Am J Case Rep.

[19] Ezaki T, Hashimoto Y, Yabuuchi E (1989). Fluorometric deoxyribonucleic acid-deoxyribonucleic acid hybridization in microdilution wells as an alternative to membrane filter hybridization in which radioisotopes are used to determine genetic relatedness among bacterial strains. Int J Syst Bacteriol.

[20] Tortoli E, Kohl TA, Brown-Elliott BA, Trovato A, Leão SC, Garcia MJ (2016). Emended description of *Mycobacterium abscessus*, *Mycobacterium abscessus* subsp. *abscessus* and *Mycobacterium abscessus* subsp. *bolletii* and designation of *Mycobacterium abscessus* subsp. *massiliense* comb. nov. Int J Syst Evol Microbiol.

[21] Nakanaga K, Sekizuka T, Fukano H, Sakakibara Y, Takeuchi F, Wada S (2014). Discrimination of *Mycobacterium abscessus* subsp. *massiliense* from *Mycobacterium abscessus* subsp. *abscessus* in clinical isolates by multiplex PCR. J Clin Microbiol.

[22] Kim BJ, Lee SH, Lyu MA, Kim SJ, Bai GH, Chae GT (1999). Identification of mycobacterial species by comparative sequence analysis of the RNA polymerase gene (rpoB). J Clin Microbiol.

[23] Yamane N, Onaga S, Saitoh H, Toyoshima S, Shimojima M, Kawahara S (2002). Multicenter evaluation of a newly developed microdilution test, broth MIC NTM to determine minimum inhibitory concentrations of antimicrobial agents for nontuberculous mycobacteria. Rinsho Byori.

[24] Clinical Laboratory Standards Institute. Susceptibility Testing of Mycobacteria, Nocardiae, and Other Aerobic Actinomycetes; Approved Standard. 2nd ed ed. CLSI document No. M24-A2. Wayne, PA: Clinical Laboratory Standards Institute; 2011. 31339680

[25] Koh WJ, Jeong BH, Kim SY, Jeon K, Park KU, Jhun BW (2017). Mycobacterial characteristics and treatment outcomes in *Mycobacterium abscessus* lung disease. Clin Infect Dis.

[26] Chung MJ, Lee KS, Koh WJ, Lee JH, Kim TS, Kwon OJ (2005). Thin-section CT findings of nontuberculous mycobacterial pulmonary diseases: comparison between *Mycobacterium avium-intracellulare* complex and *Mycobacterium abscessus* infection. J Korean Med Sci.

[27] Bass SN, Ailani RK, Shekar R, Gerblich AA (1998). Pyogenic vertebral osteomyelitis presenting as exudative pleural effusion: a series of five cases. Chest.

[28] Kurashima A (2014). Diagnosis and treatment of *mycobacterium abscessus* lung disease. Igakunoayumi..

[29] Orihashi T, Yatera K, Matsuo M, Itoh H, Yaguchi T, Miyazaki S (2012). A case of successfully treated pulmonary *Mycobacterium abscessus* infection asociated with empyema thoracies. Nihon Kokyuki Gakkai Zasshi.

[30] Nash K. A., Brown-Elliott B. A., Wallace R. J. (2009). A Novel Gene, erm(41), Confers Inducible Macrolide Resistance to Clinical Isolates of Mycobacterium abscessus but Is Absent from Mycobacterium chelonae. Antimicrobial Agents and Chemotherapy.

[31] Choi GE, Shin SJ, Won CJ, Min KN, Oh T, Hahn MY (2012). Macrolide treatment for *Mycobacterium abscessus* and *Mycobacterium massiliense* infection and inducible resistance. Am J Respir Crit Care Med.

[32] Bastian S, Veziris N, Roux AL, Brossier F, Gaillard JL, Jarlier V (2011). Assessment of clarithromycin susceptibility in strains belonging to the *Mycobacterium abscessus* group by *erm*(41) and *rrl* sequencing. Antimicrob Agents Chemother.

